# Supplementary material for: Erythropoietin, Fibroblast Growth Factor 23, and Death After Kidney Transplantation
Source: J Clin Med. 2020 Jun 4;9(6):1737. doi: 10.3390/jcm9061737 (PMC7356141; doi:10.3390/jcm9061737)
Supplement: Supplementary file 1 [file jcm-09-01737-s001.pdf]

Supplementary information

Erythropoietin, Fibroblast Growth Factor 23, and Death after Kidney Transplantation

Supplementary Table 1. Univariable associations of variables with prospective outcomes (i.e. all-cause death and cardiovascular death).

	**All–Cause Death**	**CV Death**
	HR (95%CI)	HR (95%CI)
EPO (IU/L, per SD) ‡	1.36 (1.15–1.61) ***	1.35 (1.07–1.70) *
Age (yr)	2.51 (2.02–3.13) ***	2.67 (1.95–3.65) ***
Male sex (yes vs. no)	0.91 (0.64–1.30)	0.87 (0.53–1.42)
Body surface area (m^2, per SD)	0.96 (0.80–1.15)	0.91 (0.71–1.16)
Time since renal Tx (yrs, per SD)	1.10 (0.93–1.31)	1.14 (0.90–1.45)
eGFR (ml/min/1.73 m^2, per SD)	0.66 (0.55–0.80) ***	0.70 (0.53–0.91) **
Proteinuria (≥0.5 g/24 h) (yes vs. no)	1.16 (0.78–1.72)	1.07 (0.61–1.87)
Diabetes mellitus (yes vs. no)	2.43 (1.65–3.56) ***	2.70 (1.60–4.57) ***
Systolic blood pressure (mmHg, per SD)	1.61 (1.37–1.89) ***	1.64 (1.32–2.05) ***
Total cholesterol (mmol/L, per SD)	1.00 (0.83–1.20)	1.00 (0.77–1.29)
ACE–inhibitors (yes vs. no)	0.79 (0.53–1.18)	1.04 (0.62–1.77)
Calcinurin inhibitors (yes vs. no)	0.82 (0.54–1.25)	1.08 (0.58–2.03)
Proliferation inhibitors (yes vs. no)	0.66 (0.45–0.96) *	0.47 (0.28–0.77) **
FGF23 (RU/mL, per SD)	1.77 (1.52–2.06) ***	1.78 (1.43–2.20) ***

ACE-inhibitors; angiotensin converting enzyme-inhibitors; CV, cardiovascular; eGFR, estimated glomerular filtration rate; EPO, erythropoietin; FGF23, fibroblast growth factor 23. ‡Reported hazard ratios in this Table are reported as expressed per standard deviation, whereas in the manuscript reported hazard ratios are expressed per one increase of IU/L. * <0.05, ** <0.01, *** <0.001.

Supplementary Table 2. Reporting of all hazard ratios of all covariates included in the Cox Regression Analyses for the association between erythropoietin as continuous variable and risk of all-cause and cardiovascular death (according to model 5 (including FGF23)).

	**All–Cause Death**	**CV Death**
	HR (95%CI)	HR (95%CI)
EPO (IU/L, per SD) ‡	1.15 (0.93–1.42)	1.23 (0.91–1.66)
Age (yrs, per SD)	2.16 (1.71–2.73) ***	2.34 (1.68–3.27) ***
Male sex (yes vs. no)	1.38 (0.85–2.23)	1.41 (0.72–2.76)
Body surface area (m^2, per SD)	0.77 (0.60–0.99) *	0.68 (0.48–0.98) *
Time since renal Tx (yrs, per SD)	0.94 (0.74–1.20)	0.99 (0.70–1.38)
eGFR (ml/min/1.73 m^2, per SD)	0.91 (0.71–1.16)	0.95 (0.67–1.33)
Proteinuria (≥0.5 g/24 h) (yes vs. no)	1.07 (0.70–1.65)	1.06 (0.58–1.95)
Diabetes mellitus (yes vs. no)	1.80 (1.18–2.75) **	1.89 (1.06–3.39) *
Systolic blood pressure (mmHg, per SD)	1.22 (1.02–1.46) *	1.21 (0.95–1.54)
Total cholesterol (mmol/L, per SD)	0.97 (0.80–1.18)	0.96 (0.74–1.24)
ACE–inhibitors (yes vs. no)	0.88 (0.58–1.35)	1.23 (0.69–2.19)
Calcinurin inhibitors (yes vs. no)	0.76 (0.41–1.38)	1.06 (0.43–2.62)
Proliferation inhibitors (yes vs. no)	0.74 (0.47–1.18)	0.61 (0.33–1.13)
FGF23 (RU/mL, per SD)	1.51 (1.23–1.85) ***	1.55 (1.16–2.09) **

ACE-inhibitors; angiotensin converting enzyme-inhibitors; CV, cardiovascular; eGFR, estimated glomerular filtration rate; FGF23, fibroblast growth factor 23. ‡ Reported hazard ratios in this Table are reported as expressed per standard deviation, whereas in the manuscript reported hazard ratios are expressed per one increase of IU/L. * <0.05, ** <0.01, *** <0.001.

Supplementary Table 3. Reporting of all hazard ratios of all covariates included in the Cox Regression Analyses for the association between quartiles of erythropoietin and risk of all-cause and cardiovascular death (according to model 5 (including FGF23)).

	**All–Cause Death**	**CV Death**
	HR (95%CI)	HR (95%CI)
EPO (IU/L) 1st quartile	1.00	1.00
EPO (IU/L) 2nd quartile	1.63 (0.89–3.01)	2.90 (1.22–6.91) *
EPO (IU/L) 3rd quartile	1.41 (0.77–2.57)	1.65 (0.66–4.08)
EPO (IU/L) 4th quartile	1.55 (0.82–2.91)	2.47 (0.97–6.31)
Age (yrs, per SD)	2.16 (1.71–2.74) ***	2.41 (1.72–3.38) ***
Male sex (yes vs. no)	1.36 (0.84–2.19)	1.39 (0.70–2.77)
Body surface area (m^2, per SD)	0.77 (0.60–0.99) *	0.66 (0.46–0.95) *
Time since renal Tx (yrs, per SD)	0.94 (0.74–1.19)	0.98 (0.70–1.38)
eGFR (ml/min/1.73 m^2, per SD)	0.92 (0.72–1.17)	0.93 (0.66–1.31)
Proteinuria (≥0.5 g/24 h) (yes vs. no)	1.07 (0.69–1.65)	1.02 (0.55–1.91)
Diabetes mellitus (yes vs. no)	1.78 (1.17–2.70) **	1.95 (1.09–3.49) *
Systolic blood pressure (mmHg, per SD)	1.22 (1.02–1.46) *	1.20 (0.94–1.53)
Total cholesterol (mmol/L, per SD)	0.97 (0.80–1.17)	0.96 (0.75–1.23)
ACE–inhibitors (yes vs. no)	0.90 (0.59–1.38)	1.33 (0.75–2.37)
Calcinurin inhibitors (yes vs. no)	0.76 (0.41–1.38)	1.08 (0.43–2.68)
Proliferation inhibitors (yes vs. no)	0.74 (0.47–1.18)	0.60 (0.32–1.12)
FGF23 (RU/mL, per SD)	1.56 (1.27–1.91) ***	1.62 (1.21–2.17) **

ACE-inhibitors; angiotensin converting enzyme-inhibitors; BSA, body surface area; CV, cardiovascular; eGFR, estimated glomerular filtration rate; FGF23, fibroblast growth factor 23. *<0.05, **<0.01, ***<0.001.

Supplementary Table 4. Reporting of all hazard ratios of all covariates included in the Cox Regression Analyses for the association between FGF23 as continuous variable and risk of all-cause and cardiovascular death (multivariable excluding erythropoietin (Supplemental Table 2 includes erythropoietin)).

	**All–Cause Death**	**CV Death**
	HR (95%CI)	HR (95%CI)
FGF23 (RU/mL, per SD) ‡	1.58 (1.31–1.91) ***	1.67 (1.27–2.19) ***
Age (yrs, per SD)	2.15 (1.71–2.71) ***	2.31 (1.66–3.22) ***
Male sex (yes vs. no)	1.29 (0.81–2.06)	1.28 (0.66–2.48)
Body surface area (m^2, per SD)	0.80 (0.63–1.03)	0.73 (0.51–1.03)
Time since renal Tx (yrs, per SD)	0.95 (0.75–1.20)	0.99 (0.70–1.39)
eGFR (ml/min/1.73 m^2, per SD)	0.95 (0.75–1.19)	1.00 (0.73–1.39)
Proteinuria (≥0.5 g/24 h) (yes vs. no)	1.08 (0.70–1.66)	1.07 (0.58–1.96)
Diabetes mellitus (yes vs. no)	1.73 (1.14–2.63) **	1.79 (1.00–3.20) *
Systolic blood pressure (mmHg, per SD)	1.24 (1.04–1.49) *	1.23 (0.97–1.58)
Total cholesterol (mmol/L, per SD)	0.96 (0.79–1.16)	0.94 (0.72–1.22)
ACE–inhibitors (yes vs. no)	0.85 (0.56–1.29)	1.14 (0.65–2.01)
Calcinurin inhibitors (yes vs. no)	0.72 (0.39–1.30)	0.96 (0.39–2.39)
Proliferation inhibitors (yes vs. no)	0.77 (0.48–1.22)	0.64 (0.34–1.21)

ACE-inhibitors; angiotensin converting enzyme-inhibitors; CV, cardiovascular; eGFR, estimated glomerular filtration rate; FGF23, fibroblast growth factor 23 ‡Reported hazard ratios in this Table are reported as expressed per standard deviation, whereas in the manuscript reported hazard ratios are expressed per one increase of RU/mL. * <0.05, ** <0.01, *** <0.001.

Supplementary Table 5. Prevalence of the different etiologies of CKD described in the total cohort of 579 RTRs and across the quartiles of erythropoietin levels.

**Total Cohort**		**Quartiles of EPO**				***P*-value**
**Etiology of CKD (*n*,%)**	**Total**	**Q1**	Q2	Q3	Q4	0.005
Primary glomerular disease	160 (28)	45 (31)	47 (33)	40 (28)	28 (19)	0.05
Glomerulonephritis	38 (7)	17 (11)	4 (3)	9 (6)	8 (6)	0.02
Tubular Interstitial Disease	90 (16)	17 (12)	30 (21)	27 (19)	16 (11)	0.04
Polycystic Renal Disease	104 (18)	15 (10)	22 (15)	26 (18)	41 (28)	0.001
Dysplasia and Hypoplasia	31 (4)	5 (3)	6 (4)	4 (3)	6 (4)	0.90
Renovascular Disease	32 (6)	12 (8)	5 (4)	7 (5)	8 (6)	0.35

Diabetic Nephropathy	23 (4)	7 (5)	4 (3)	4 (3)	8 (6)	0.52
Other or unknown cause	111 (19)	28 (19)	25 (18)	28 (19)	30 (21)	0.92

CKD, chronic kidney disease; EPO, erythropoietin.
